# Response to Combination of Pembrolizumab and Axitinib in Hereditary Leyomiomatosis and Renal Cell Cancer (HLRCC)

**DOI:** 10.3390/curroncol28040216

**Published:** 2021-06-25

**Authors:** Ibon Gurruchaga Sotés, Ana Nuño Alves, Sandra Vicente Arregui, Carmen Santander Lobera

**Affiliations:** 1 Medical Oncology Department, Miguel Servet University Hospital, 50009 Zaragoza, Spain; csantanderl@salud.aragon.es; 2 Medical Oncology Department, Obispo Polanco Hospital, 440002 Teruel, Spain; anunno@salud.aragon.es; 3 Pathology Department, Miguel Servet University Hospital, 50009 Zaragoza, Spain; svicentea@salud.aragon.es

**Keywords:** hereditary leyomiomatosis, renal cell cancer, pembrolizumab, axitinib

## Abstract

In current clinical guidelines, such as those provided by the National Comprehensive Cancer Network (NCCN), evidence for treatment is based on a small clinical trial that included patients with HLRCC. They support the use of the combination of erlotinib and bevacizumab as the first therapeutic option in this rare condition. In the present study, we report a rare case of this condition in an 18-year-old male with a family history of kidney cancer whom we successfully treated with surgery and a novel drug treatment modality based on the combination of an immune check-point inhibitor (ICPI) and a tyrosine-kinase inhibitor (TKI) with excellent and promising results.

## 1. Introduction

Hereditary leyomiomatosis and renal cell cancer (HLRCC) is an autosomal dominant genodermatosis of variable penetrance associated with a mutation of the gene encoding for fumarate hydratase (FH) at the level of 1q chromosome, in which missense mutations are the most common. This gene codes for an enzyme that catalyzes the reversible hydration/dehydration of fumarate to malate in the tricarboxylic acid cycle. Homozigous patients for the mutation may suffer fumaric aciduria, progressive encephalopathy, hypotonia and epileptic seizures, and do not survive beyond a few months of life.

Nevertheless, patients who are heterozygous for the mutation can develop multiple cutaneous piloleiomyomas, fibroids and have a 15% accumulated risk of type II papillary renal cell carcinoma (RCC). High concentrations of fumarate, secondary to the deficit of FH, leads to the inhibition of the hydroxylase of hypoxia inducible factors (HIF), therefore, an accumulation of HIF and an increase in downstream transcription factors related to cell proliferation, cell survival, and angiogenesis through the VEGF and GLUT1 pathways [1,2].

Using genomic mapping, Alam et al., described a minimum interval that contained the locus of the gene, which they called MCUL1 (multiple cutaneous and uterine leiomyomata), a region of 14 cM that can act as a tumor suppressor gene [3]. To date, three large series of cases have been reported and their mutation patterns have been analyzed [4,5,6]. Gardie’s study identified up to 32 mutations for the FH gene, including 21 mutations not previously described.

Recently, Shuch et al. (2020) studied data sets from the 1000 Genomes Project (1000GP) and the Exome Aggregation Consortium (ExAC) containing sequencing data for the FH gene. They concluded that FH alterations are carried by 1 in 1000 patients with renal cell cancer and its penetrance varies between 1.7% and 5.8%, lower than estimated in other series [7].

We report a case of an intervened advanced RCC associated with HLRCC managed with a multimodal approach, combining surgery followed by systemic treatment that includes the combination of pembrolizumab and axitinib with promising results not described before in the literature and based on the pseudo-hypoxic status and a pro-inflammatory condition of the tumor.

## 2. Case Report

**Figure 1:**
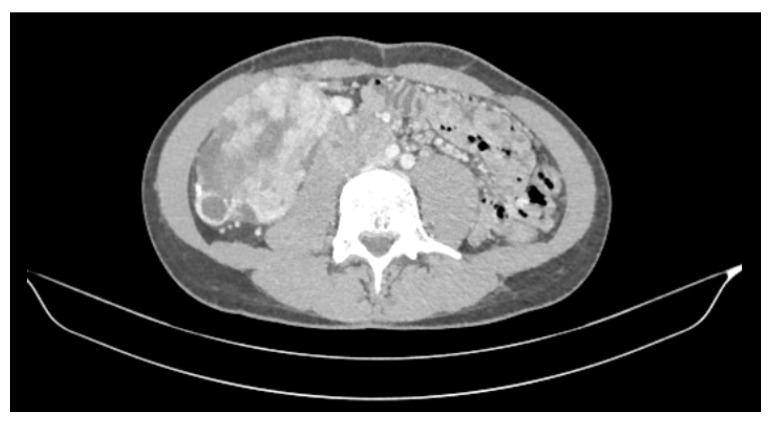
CT at diagnosis. Mass at the upper pole of the right kidney.

We present an 18-year-old male with medical history of generalized epilepsy treated with lacosamide 100 mg twice a day and family history of a grandfather and an uncle diagnosed with and dead of renal cancer, both at the age of 40. The first presenting symptoms were haematuria and loss of 18 kg of weight in 3 months. The patient is diagnosed with a mass at the upper pole of the right kidney with a size of 10.5 × 6 × 9 cm, a retroperitoneal conglomerate of 7 × 3 cm with inferior vena cava infiltration, thrombus at the right renal artery, and an enlarged adenopathy in the aortic bifurcation of 4.6 × 2.8 cm (Figure 1).

**Figure 2:**
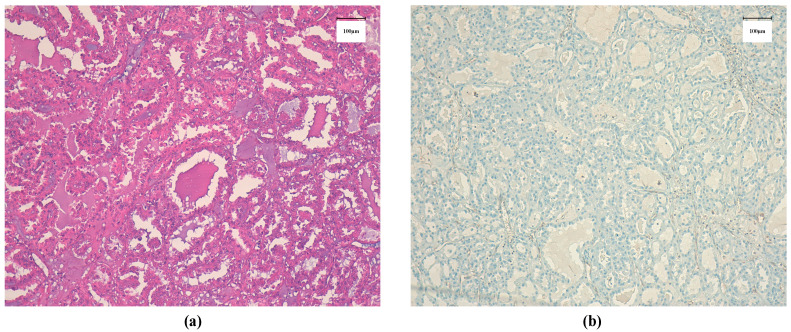
Biopsy showing papillary and tubular renal carcinoma (**a**): Histological section with hematoxylin-eosin stain showing renal cell carcinoma with eosinophilic nucleolus surrounded by perinuclear halo. (**b**): Histological section showing loss of expression of FH. Scale bar 100 µm.

A stereotactic biopsy was performed with the result being papillary and tubular renal carcinoma. He underwent a radical right nephrectomy with extended retroperitoneal, retrocaval and periaortic lymphadenectomy. The pathological result shows a renal cell carcinoma with papillary, tubular, tubulocistyc, and solid pattern, with an affected vascular margin and with suspicion of fumarate hydratase (FH) loss of expression due to the presence of an eosinophilic nucleolus surrounded by a perinuclear halo (Figure 2a,b). TNM stage: pT3a pN1 (four out of seven lymph nodes affected).

**Figure 3:**
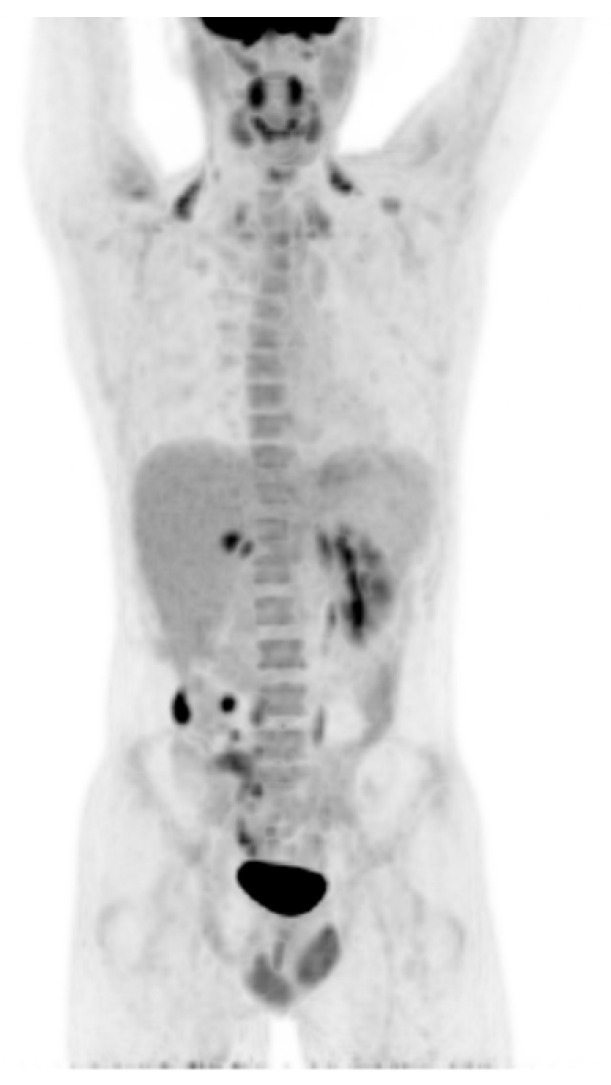
PET-CT confirming first progression, with locoregional lesions, hepatic and retroperitoneal metastasis.

After recovery, a positron emission tomography/computed tomography.

(PET/CT) scan was performed, showing disease persistence at lateroconal fascia, right psoas, peritoneum, retroperitoneum and a metastatic lesion in the hepatic VII segment (Figure 3). The patient was also submitted to genetic mapping and genetic counselling, identifying a deletion of 3.9 kB in the 1q43 chromosomal region, which includes exon 8 of the FH gen in heterozygosity, associated with hereditary leiomyomatosis and renal cell cancer (HLRCC).

**Figure 4:**
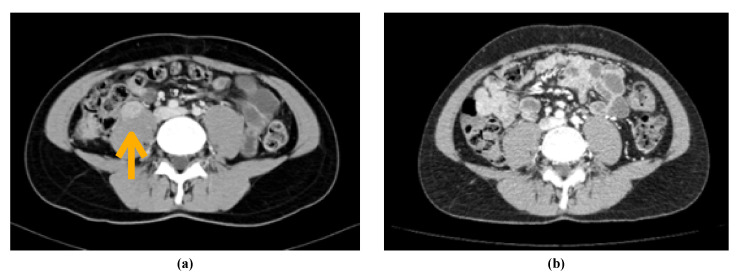
CT scans showing the evolution of the oncological disease. (**a**): CT confirming second progression. Increase of retroperitoneal node and a new node anterior to the right psoas. (**b**): CT with major partial response. Shows stable retroperitoneal node and resolution of the rest of metastatic disease.

The patient started biweekly bevacizumab (10 mg/m^2^) and erlotinib 150 mg QD. He developed grade 2 hypothyroidism, which was treated with levothyroxine oral supplementation and grade 3 acneiform rash requiring erlotinib interruption and topic treatment. At the two-month follow up visit, the patient presented radiological disease progression according to RECIST 1.1 criteria. A retroperitoneal node size increase of up to 32 × 24 mm and a new node at the anterior surface of the right psoas of 23 × 16 mm (Figure 4a). With ECOG (Eastern Cooperative Oncology Group) 1, mild abdominal pain and grade 1–2 rash, the patient started the second line of treatment with pembrolizumab 200 mg every 3 weeks combined with axitinib 5 mg BID. After 2 months, the patient’s abdominal pain resolved, and the CT scan revealed an important partial response to the therapy with a reduction of the retroperitoneal node and the disappearance of the other lesions (Figure 4b). Currently, the patient is undergoing the 11th cycle of treatment, with current toxicity being grade 1–2 diarrhea managed with loperamide and requiring temporary interruptions of axitinib. He has reached an overall survival of 20 months and a disease free survival of 15 months, with persistent good response and improved symptom control, since beginning treatment with pembrolizumab-axitinib.

Investigators obtained written consent from the patient for publishing this case report, including medical history, images, and treatments received.

## 3. Discussion

HLRCC is a rare hereditary condition with no more than 300 families reported worldwide. It is considered an orphan disease with no high-level evidence available or specific clinical trials designed for this disorder. The available treatment options for this condition in the metastatic setting are supported by the results of a few cases gathered from larger trials on kidney cancer. Based on the NCT01130519 clinical trial, where 42 out of 83 patients had HLRCC, NCCN guidelines recommend the combination of bevacizumab and erlotinib as the first line treatment for the metastatic scenario. This trial showed an overall response rate (ORR) of 64%, with progression free survival (PFS) of 21 months, and grade 3–4 toxicities of 47% [8].

In the Keynote426 clinical trial, the combination of axitinib and pembrolizumab in RCC has encouraging results with an ORR of 59.3% and a PFS of 15 months independent of PDL1 expression and International Metastatic Renal Cell Carcinoma Database Consortium risk groups [9]. For the use of the combination in the case reported, we relied on the pseudo-hypoxic status triggered by FH deficiency, as well as a pro-inflammatory condition demonstrated by a retrospective study in which a higher proportion of PDL1 overexpression was observed in the subgroup of patients with HLRCC, together with a greater number of PD1-expressing CD8 tumor-infiltrating lymphocytes at the tumor margins. The authors concluded that these patients could present a greater response to immune check-point inhibitors [ICPI] [10].

Recently, the first two cases of stage IV HLRCC treated with a combination of ICPI and TKI have been published, using the combination of nivolumab-axitinib and sintilimab-axitinib, showing promising results with sustained partial responses [11,12].

## 4. Conclusions

This case reports a young patient with a rare hereditary condition, HLRCC. It underlines the need to look for genetic alterations in young patients with kidney tumors (especially if they present a family history). Moreover, it highlights that adequate genetic profiling/counselling should be available in these cases in order to identify specific mutations and syndromes. Finally, this case report proposes a novel treatment strategy based on a combination of surgery, pembrolizumab, and axitinib, with promising results. The newly established European Reference Network (ERN) eUROGEN, is currently working on building online platforms to discuss rare urological diseases, such as the one presented here, update and adapt current guidelines, and offer physicians and patients a unique opportunity to manage complex and rare conditions with an specific workstream flow of rare urogenital tumors, such as HLRCC, with more than 4000 patients managed per year in those ERN eUROGEN centers [13].

## Data Availability

The data presented in this study are available in this article.
